# Time-Frequency Spectral Signature of Limb Movements and Height Estimation Using Micro-Doppler Millimeter-Wave Radar

**DOI:** 10.3390/s20174660

**Published:** 2020-08-19

**Authors:** Yael Balal, Nezah Balal, Yair Richter, Yosef Pinhasi

**Affiliations:** Faculty of Engineering, Ariel University, Ariel 40700, Israel; nezahb@ariel.ac.il (N.B.); yairr@ariel.ac.il (Y.R.); yosip@ariel.ac.il (Y.P.)

**Keywords:** human catalog targets, micro-Doppler radar, millimeter-wave radar

## Abstract

We present a technique for the identification of human and animal movement and height using a low power millimeter-wave radar. The detection was based on the transmission of a continuous wave and heterodyning the received signal reflected from the target to obtain micro-Doppler shifts associated with the target structure and motion. The algorithm enabled the extraction of target signatures from typical gestures and differentiated between humans, animals, and other ‘still’ objects. Analytical expressions were derived using a pendulum model to characterize the micro-Doppler frequency shifts due to the periodic motion of the human and animal limbs. The algorithm was demonstrated using millimeter-wave radar operating in the W-band. We employed a time–frequency distribution to analyze the detected signal and classify the type of targets.

## 1. Introduction

Millimeter-wave (MMW) radar is becoming increasingly commercial and applicable due to recent technological developments. This radar is employed as a detection measure in many applications, such as collision avoidance radars in automobiles [1,2] and stand-off remote sensors in homeland security applications [3]. Additional developments are required in order to differentiate between targets, and in particular, to distinguish between a person and an animal or other objects.

Detecting a target with a Doppler MMW radar has several advantages compared to other remote sensing technologies, primarily in bad weather conditions and stationary background clutter scenarios [4]. The micro-Doppler target signature is considered one option for automatic target recognition and classification (ATR) systems [5]. The micro-Doppler target signature is a time-varying frequency shift caused by the relative movement of separate parts of the target, which can be studied to extract additional information on target characteristics [6].

Several works exploiting micro-Doppler signatures for target classification and human identification have been published [7,8,9,10,11,12,13,14,15,16,17,18,19,20]. In this paper, we focused on the possibility of characterizing object movement and differences between targets. The approach presented here is based on the analysis of a physical pendulum motion signature, which resembles human limb motion. The detected periodic limb gestures are associated with physical pendulum oscillations, enabling the evaluation of limb lengths. Micro-Doppler time–frequency variations can even be employed to estimate the height of a person, thus, increasing the reliability of target recognition. This technique was demonstrated using a MWW high-resolution radar operating at 94 GHz.

This paper is organized as follows: In Section 2, we describe the principles of micro-Doppler radar operation in relation to the detection of periodical target movement signatures via the physical pendulum motion model. The experimental setup is presented in Section 3. In Section 4, we show the time–frequency signals obtained from physical pendulum oscillations. In Section 5, we compare the detected radar signals of human and animal motions with a video movie taken in the arena, demonstrating the differences between their corresponding micro-Doppler signals. In Section 6, we summarize and conclude the paper.

## 2. Target Characterization Using Micro-Doppler Radar

**Figure 1:**
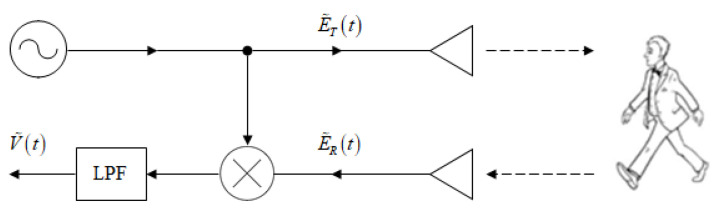
Continuous wave (CW) micro-Doppler radar.

The principal scheme of a continuous wave (CW) micro-Doppler radar is shown in Figure 1. The transmitted signal is a CW millimeter-wave at a constant carrier frequency $f_{0}$:

(1) $${\tilde{E}}_{T}\left(t\right)=A_{T}e^{j2\pi f_{0}t}$$

Scattered by the target, the reflected signal received by the radar is:

(2) $${\tilde{E}}_{R}\left(t\right)=A_{R}e^{-j[2k\cdot r(t)-\theta ]}\cdot e^{j2\pi f_{0}t}$$

where $A_{T}$ and $A_{R}$ are the amplitudes of the transmitted and received signals, respectively, $k=2\pi f_{0}/c$ is the wavenumber (*c* is the speed of light), r(t) is the distance to the moving target, and θ is a constant phase shift. The detection is based on heterodyning the reflected signal (2) with the transmitted CW carrier (1), resulting in the product:

(3) $$\tilde{V}\left(t\right)={\tilde{E}}_{T}\left(t\right)\cdot {\tilde{E}}_{R}^{*}\left(t\right)=A_{T}A_{R}e^{j[2k\cdot r(t)-\theta ]}$$

Inspection of Equation (3) reveals a time varying phase:

(4) φ(t)=2k·r(t)−θ

The radial velocity of the target respective to the radar is given by the time derivative of the location r(t) according to:

(5) $$\frac{d}{dt}r\left(t\right)=\dot{r}\left(t\right)=v_{r}\left(t\right)$$

Using Equations (4) and (5), the instantaneous Doppler frequency shift can be derived:

(6) $$f_{d}\left(t\right)=\frac{1}{2\pi }\frac{\partial \varphi (t)}{\partial t}=\frac{1}{2\pi }2k\cdot \dot{r}\left(t\right)=\frac{2f_{0}}{c}\cdot v_{r}\left(t\right)$$

The instantaneous Doppler frequency $f_{d}\left(t\right)$, obtained at the product detector output, is proportional to the carrier frequency $f_{0}$. Thus, in the MMW regime, the micro-Doppler frequency shift is revealed more distinctly. Increasing $f_{0}$ results in a higher instantaneous micro-Doppler deviation of $f_{d}\left(t\right)$ at the IF, due to body parts (limbs) movement.

**Figure 2:**
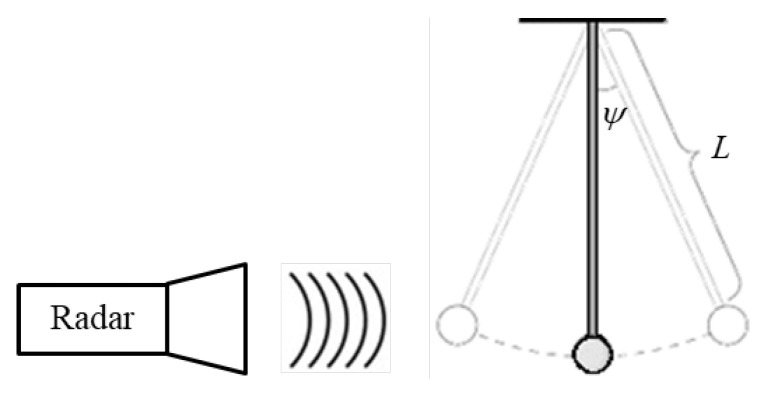
Illustration of the detection of a physical pendulum oscillation.

Human hand and leg motions can be regarded as non-uniform oscillations, expressed in the radar IF signal via instantaneous periodic frequency deviations due to the micro-Doppler effect. They can be treated like the motion of a physical pendulum. We now demonstrate how the pendulum length can be calculated from the instantaneous frequency Equation (6). For convenience, we assume that the radar is directed to the oscillating pendulum, as illustrated in Figure 2.

The angular displacement of a physical pendulum can be represented by:

(7) $$\psi \left(t\right)=\psi_{max}\sin\left(\frac{2\pi }{T}t\right)$$

where *T* is the temporal period of the oscillation, and $\psi_{max}$ is the initial pendulum angle. The angular velocity of the physical pendulum is given by the time derivative:

(8) $$\dot{\psi }\left(t\right)=\frac{2\pi }{T}\psi_{max}\cos\left(\frac{2\pi }{T}t\right)$$

resulting in a radial velocity:

(9) $$v_{r}\left(t\right)=L\cdot \dot{\psi }\left(t\right)=v_{max}\cos\left(\frac{2\pi }{T}t\right)$$

where *L* is the length of the physical pendulum and $v_{max}$ is the maximum radial velocity obtained at $\psi =0^{\circ }$. The length of the physical pendulum can be found from Equations (8) and (9) via the relation:

(10) $$L=\frac{v_{r}\left(t\right)}{\dot{\psi }\left(t\right)}=\frac{1}{2\pi }\frac{v_{max}}{\psi_{max}}\cdot T$$

Equation (10) indicates that, if measuring the maximum radial velocity $v_{max}$, the maximum pendulum angle $\psi_{max}$, and the period time *T*, one can calculate the physical pendulum length *L*. The model here assumes harmonic motion, oscillating not necessarily at a ‘natural’ frequency of the pendulum. When external forces are introduced, they determine the period, time *T*, which, in general, may be different from the resonance pendulum frequency. In the following, we perform this technique of calculating *L* by evaluating $v_{max}$, $\psi_{max}$, and *T*. The radial velocity $v_{r}\left(t\right)$ of the moving pendulum is measured using a Doppler CW radar operating in the W-band. To follow the periodic structure of the resulting frequency deviations, it is important that the Doppler shift $f_{d}\left(t\right)$ be sufficiently high, i.e., $f_{d}\left(t\right)\gg 1/T$.

As noted, according to Equation (6), the intermediate frequency $f_{d}\left(t\right)$ is proportional to the carrier frequency $f_{0}$. Utilizing a carrier in extremely high frequencies (EHF) results in a relatively high micro-Doppler shift $f_{d}\left(t\right)$ in the kHz regime. This enables revealing in the spectrogram the frequency variations due to the limbs movement. Although the movement of the limbs is not necessarily a pure sine wave, it is periodic with a fundamental frequency 1/T, which can be identified from the detected signal.

## 3. Experimental Setup

**Figure 3:**
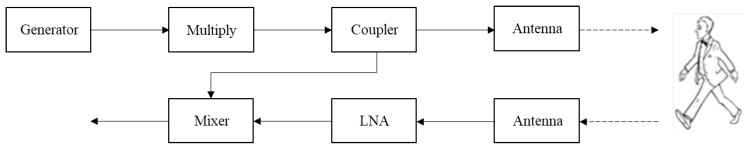
A scheme of the CW micro-Doppler radar.

A schematic illustration of the MMW radar is shown in Figure 3. An RF signal generator is producing a CW signal at 15.67 GHz. The signal frequency is upconverted to the W-band regime by a×6 multiplier, resulting in a 94 GHz CW carrier. 10% of the output signal is coupled out to a mixer serving as a product detector. The transmitting and receiving antennas are both directive horn-lens antennas, with a gain of 30 dBi. The signal scattered from the moving target is amplified by a 30 dB low noise amplifier (LNA), with a noise figure of 5 dB. The IF signal from the product detector is digitized by an analog to digital converter (A/D). The sampling rate was set to 10 ksample/s sufficient for identifying micro-Doppler frequency shifts.

**Figure 4:**
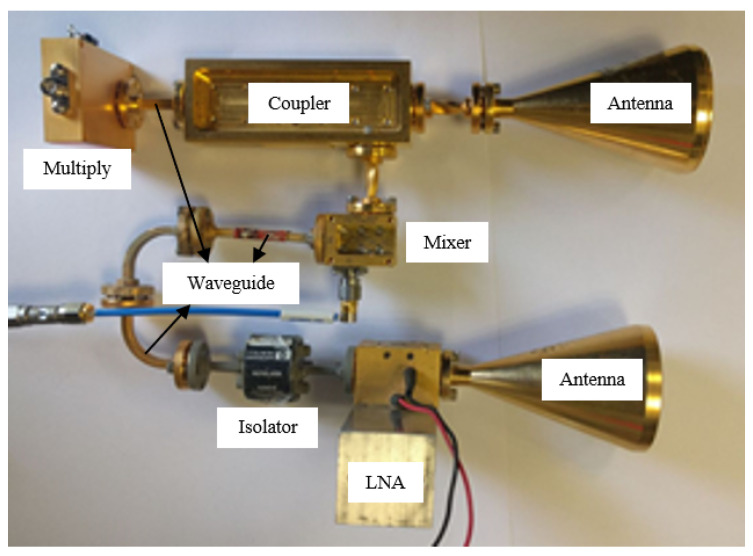
The CW micro-Doppler radar.

Operating at 94 GHz enables the utilization of small aperture directive antennas resulting in the detection of small targets with low radar cross-section (RCS). At 94 GHz, the atmospheric attenuation is low (atmospheric transmission window). This enables increasing the distance to the target, enabling detection even in adverse weather conditions. The distance between the radar and the detected moving object was between 3 to 25 m. A photo of the radar is given in Figure 4.

**Figure 5:**
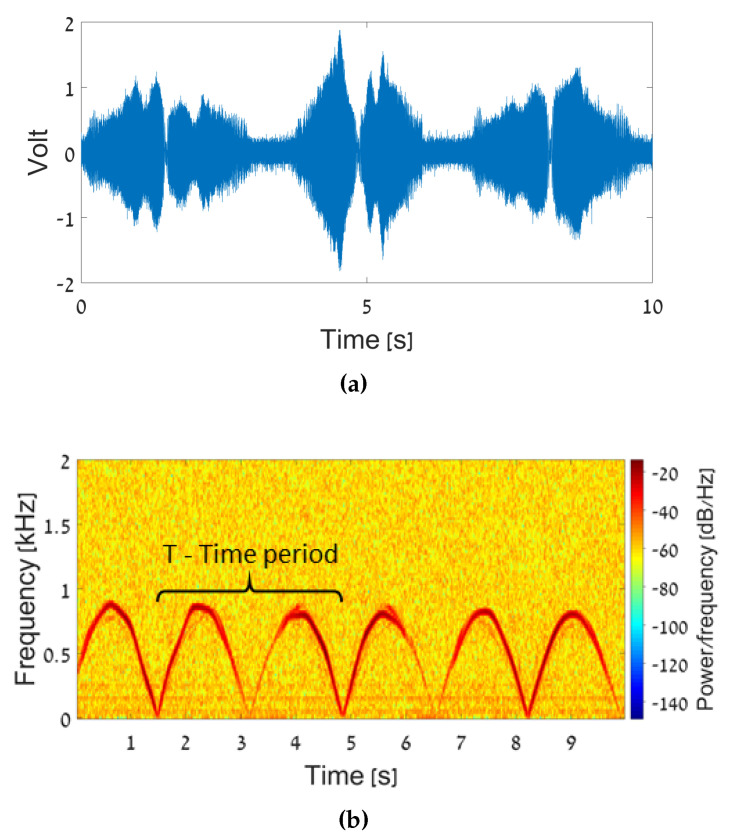
The measurement results with the physical pendulum: (**a**) The intermediate frequency (IF) signal in the time domain. (**b**) Spectrogram of the micro-Doppler frequencies.

In preliminary experiments, we studied the motion of a physical pendulum via the micro-Doppler signature obtained at the output of the product detector in the scenario illustrated in Figure 2. We examined three pendulums with different lengths, each starting to oscillate from two initial angles, i.e., $\psi_{max}=10^{{}^{\circ }}$ or $20^{{}^{\circ }}$. The IF signal resulted from the pendulum motion is shown in the time domain in Figure 5a. In order to generate its spectrogram (see Figure 5b), a discrete fast Fourier transformation (FFT) is employed with a short temporal window. The spectrogram in Figure 5b shows the instantaneous frequency as a function of time. The intensity of the detected signal is presented by color; blue represents low-level intensities while red is for higher IF signal strength.

From experimental studies, we learned that for the human walking scenario, the optimal window temporal width should be about $3f_{d}$, where $f_{d}$ is the maximum expected frequency deviation. The window should be long enough to achieve the resolution required to identify the different micro-Doppler frequency shifts, but not so long as to enable following the temporal behavior of the instantaneous frequency. In the case of an oscillating pendulum, the maximum expected frequency shift was 1 kHz. Therefore, we set the FFT window, in this case, to be 3 ms.

The presented experiments were performed using a single mixer as a product detector, resulting in positive base-band frequency deviations only. Consequently, the calculated velocities are shown in the spectrogram are in positive frequencies, even if the pendulum is moving backward. By employing quadrature I/Q radar, it will also be possible to identify the direction of movement i.e., negative velocities.

The parameters of the physical pendulum motion were analyzed using the time–frequency spectrogram. The inspection of Figure 5b reveals a cyclic structure of the micro-Doppler frequency shift, corresponding to the half pendulum oscillation period *T*. Using Equation (6), the maximum pendulum velocity $v_{max}$ was calculated from the frequency deviation in the peaks. With the substitution of $v_{max}$, $\psi_{max}$, and *T* into Equation (10), we can estimate the physical pendulum length *L*. The experimental results are summarized in Table 1. The error analysis, the length error ΔL, and the length uncertainty δL are described below in Equations (12) and (13).

The experimental results showed that the length of a pendulum could be evaluated from the micro-Doppler frequency deviation quite accurately. Errors of several percent still allowed us to identify the characterizations of the object detected by the W-band radar. In the following, we demonstrate an application of the algorithm to detect and identify when the target is a walking human.

## 4. Human Motion

The movement of human limbs can be referred to as a physical pendulum motion. Therefore, exploiting the micro-Doppler signatures of human motion enables the evaluation of limb lengths. There is a direct connection between the length of human limbs $L_{hand}$ or $L_{leg}$ and height *H* as indicated in [21] by the relation:

(11) $$H=\frac{L_{hand}}{0.44}=\frac{L_{leg}}{0.53}$$

**Figure 6:**
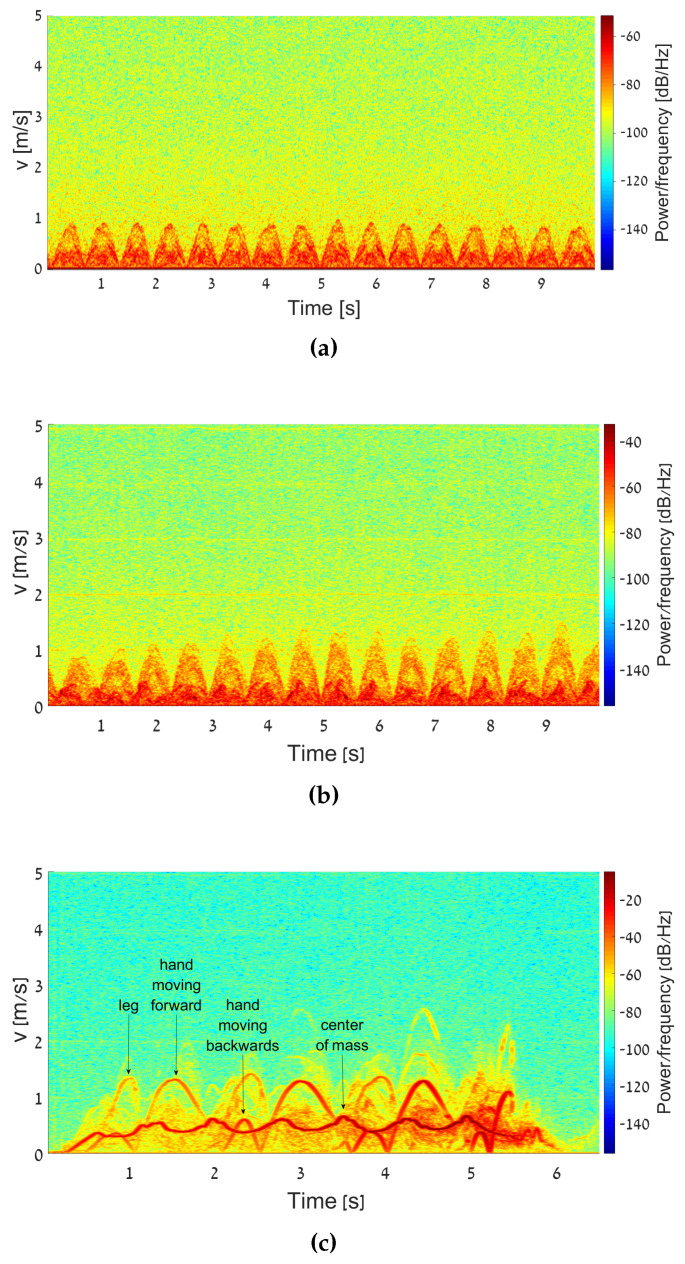
Human motion spectrogram: (**a**) Hand motion alone. (**b**) Leg motion alone. (**c**) Normal human walking.

Following [21], this formula represents the expected value of height for a given limb length. Based on a large sample apace, it is claimed to be a reliable estimation for adults and children. The micro-Doppler signatures of a walking adult were detected with the W-band radar motion, as illustrated in Figure 3. The adult height was 1.72 m. Using time–frequency analysis, we decomposed the total adult motion into separate body movements and examined the model for each of them. At the first stage, only hand motion was analyzed. The spectrogram of the resulting IF signal of a standing person moving his hands only is shown in Figure 6a. Next, the detection procedure was applied to the detection of moving legs only, neutralizing the moving hands and the shift of the center of mass. The corresponding spectrogram is shown in Figure 6b.

These two different experiments were performed in order to characterize the motions of hands and legs separately. We compared these two movements to the physical pendulum motion model, described previously, and found a high correlation. These movements were periodic, with speed increasing until it reached the peak and then fading and repeating. Finally, a measurement was conducted for normal adult walking, while three little corner reflectors were attached to hand, leg, and stomach, increasing the strength of the reflected signal at the radar receiver. The result is shown in Figure 6c, and this result consists of a superposition of the movement of the legs, hands, and center of mass.

**Figure 7:**
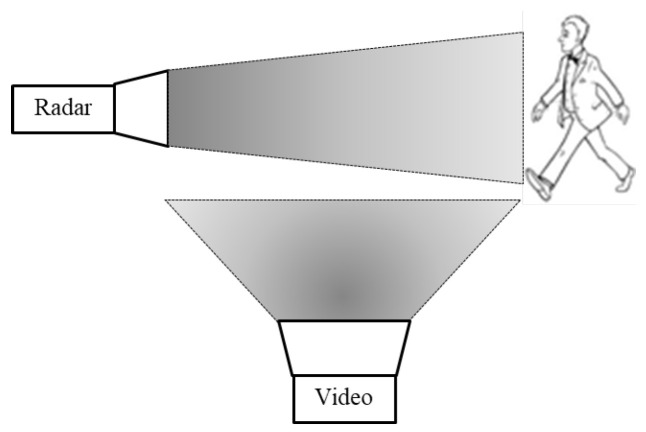
Video capturing a human walk.

**Figure 8:**
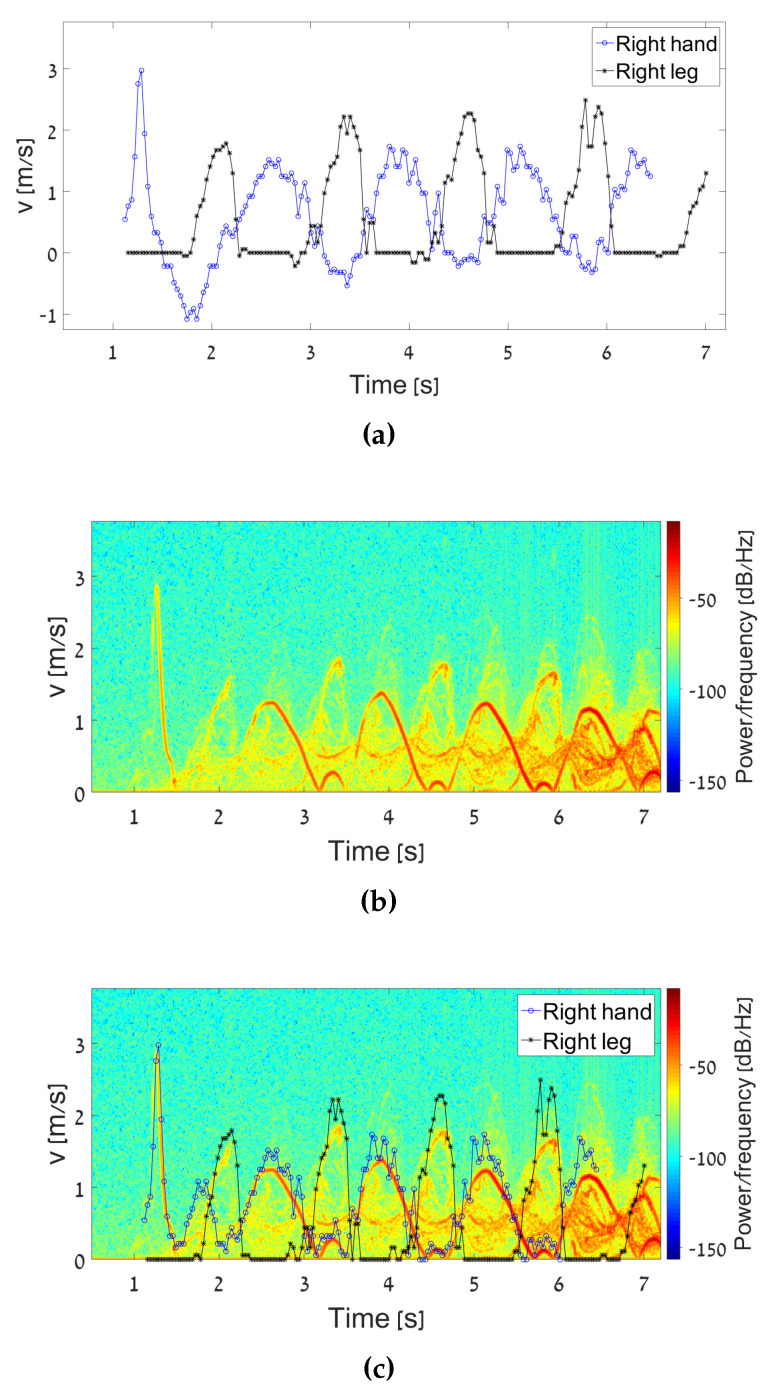
Velocity of the right hand and leg: (**a**) Obtained by video frames. (**b**) Obtained by micro-Doppler radar. (**c**) Comparison between the two methods.

In another measurement, simultaneously to the radar measurements, a video movie was captured, as illustrated in Figure 7. This was used for monitoring the experimental scene. The moving video frames were analyzed to resolve the velocity of the right hand and right leg as a function of time (see Figure 8a). The analysis was conducted using a signal processing procedure that followed the edge of the right hand and leg. Figure 8a shows both the hand and leg instantaneous velocities during the walking. We observed that the maximum speed of the leg was higher than that of the hand. The leg was longer than the hand and therefore, its radial velocity was higher. In addition, it can be seen that the periodic movement of the hand and the leg were in reverse phase, i.e., when the hand moved forward, the foot moved backward and vice versa.

An interesting phenomenon revealed from the graphs in Figure 8a is that the leg movement did not have a negative speed relative to the radar, while the hand did. This is actually a typical walking style. The instantaneous velocity of the limbs obtained from the spectrogram generated by the micro-Doppler experimental data is given in Figure 8b. Figure 8c shows a comparison between the instantaneous velocity of the limbs obtained by the video camera and by the spectrogram generated by the micro-Doppler experiment. In order to synchronize between these velocity graphs, a quick move of the right hand, at the beginning of the movement, was performed. This can be seen by the high peak at the beginning of the movement, as shown in Figure 8a,b. Figure 8c shows a clear correspondence between the velocities attained from the two measures. As mentioned, the velocities of the hand or leg moving backward are all shown in the spectrogram at positive frequencies since a single product detector was used for heterodyne detection.

Inspection of the spectrogram revealed an oscillatory frequency deviation with a period of T=1.266 s, for both leg and hand. The maximum velocity deviation $v_{max}$ of the leg was observed to be 2.5 m/s, and for the hand, 1.8 m/s. The initial angles of the limbs $\psi_{max}=30^{{}^{\circ }}$ were determined via physiological studies, characterizing the angle of the hands and legs during walking [22,23]. Using the expressions (10) and (11), the human height was evaluated. Quantitative calculations of the target characteristics are summarized in Table 2.

The height error ΔH was calculated via:

(12) $$\Delta H=\left|\frac{H_{measured}-H_{calculated}}{H_{measured}}\right|\cdot 100\%$$

and the height uncertainty δH was calculated using:

(13) $$\delta H=\sqrt{{\left(\frac{\partial L}{\partial \psi_{max}}\cdot \Delta \psi_{max}\right)}^{2}+{\left(\frac{\partial L}{\partial v_{max}}\cdot \Delta v_{max}\right)}^{2}+{\left(\frac{\partial L}{\partial T}\cdot \Delta T\right)}^{2}}$$

**Figure 9:**
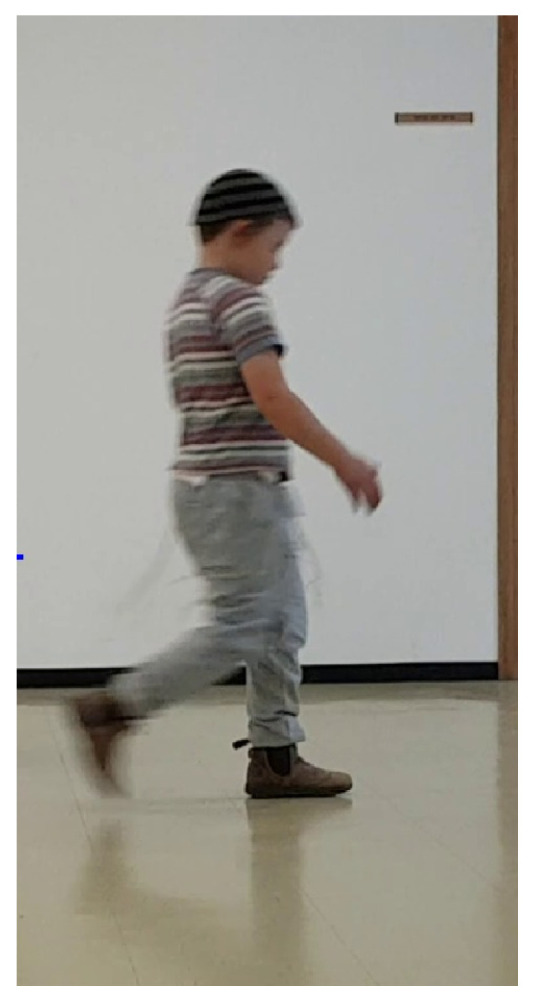
Detection of a walking child.

The micro-Doppler signatures of a walking child were also identified. The measurement method for child movement was equivalent to that employed for an adult and included micro-Doppler signal analysis combined with image processing to ensure a fit. Figure 9 shows the walking child during measurements. The results are summarized in Table 2.

Examining the results given in Table 2 revealed a variance between the height when estimating via the lengths of the limbs. Estimating the height via the leg length was more accurate than when using the measure of the hand. In both cases, an error of a few percent is still within the limit enabling to distinguish between moving targets; human adults, infants, and animals. Supplementary walking parameters, such as body and limb velocities, can further reduce uncertainties.

## 5. Animal Motion

**Figure 10:**
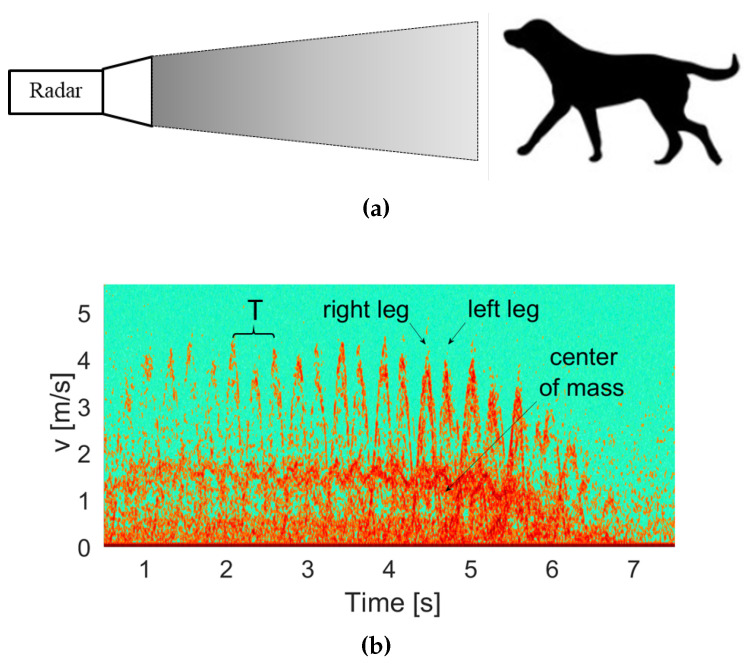
(**a**) Illustration of the detection of a walking dog. (**b**) Dog motion spectrogram.

The technique can be used to detect and identity animals. Although the RCS of a small animal may be lower than that of a human, the MMW radar can still perceive it due to its high directivity. The identification is made via the micro-Doppler typical signature of a moving animal, which is shown to be different from that of humans. The micro-Doppler signatures of a walking dog were also detected with the W-band radar, as illustrated in Figure 10a. The spectrogram generated by the micro-Doppler that resulted from the detected signal is given in Figure 10b. The signature of the dog is different from that of a human. Comparing the spectrogram of human motion as shown in Figure 8 and the resulting spectrogram of dog motion (Figure 10b) revealed that the center of mass velocities of the human was approximately 0.6 m/s and that of the dog was higher, approximately 1.6 m/s. The dog presented a higher number of steps due to its shorter limb lengths. In addition, inspection of the frequency peaks in the spectrogram showed that the maximum speed did not change and remained constant at each step. This is because the lengths of the dog’s front and rear limbs are all equal. Unlike a human, whose hand length is shorter than that of the legs and therefore their corresponding velocities are different, as revealed in the frequency peaks appearing in the spectrogram of Figure 8.

When discussing the movement of animals, we note that their radar cross-section (RCS) is smaller than the RCS of a human. Therefore, when performing radar measurements for an animal, the resulting signal-to-noise ratio (SNR) is expected to be somewhat lower. This phenomenon is observed when comparing the intensity of Figure 10b with Figure 8.

In the experiment, the height of the dog was 0.6 m and the leg length was 0.37 m. From the peaks in Figure 10b, the maximum speed was calculated to be 4 m/s. The temporal period was 0.15 s and the initial angle was 15 degrees. Using Equation (10), the length of the dog’s leg was calculated to be 0.35 m, which is a coincidence to the direct measurement.

The differences between the human and animal movement signatures, obtained from the micro-Doppler shifts, for the center of mass velocities as well as the different (hand and leg) limb velocities and their corresponding length estimations, can be used for differing between these two targets.

## 6. Summary and Conclusions

This paper demonstrated a technique for differing between targets and identifying a moving target via the micro-Doppler signature obtained using a stand-off Doppler radar operating in millimeter wavelengths. We employed a directive CW radar, operating in the W-band, to detect frequency shifts and additional instantaneous frequency deviations due to body movements. Time–frequency analysis showed that in addition to the Doppler constant frequency shift caused by the target movement, additional deviations in the frequency were realized due to the inherent motions of different body parts. These micro-Doppler instantaneous frequency shifts were periodic in time and corresponded to the unique motions of the moving targets.

We applied this approach for the identification of targets and were able to differentiate between adults, children, and animals. The micro-Doppler signatures emerging from limb motions enabled even the evaluation of the physical limb length with a high accuracy. We made a comparison between the spectrograms obtained from the Doppler radar and the signal processing of the video frames. We demonstrated that in a walking human or animal scenario, the moving velocity, as well as the target height, could be evaluated via their corresponding micro-Doppler signatures. This technique can be used to catalog targets by employing high-resolution millimeter-wave radars and can distinguish between target types, including adults, children, and different animals.

The proposed micro-Doppler technique is suggested for target identification in indoor or outdoor scenarios. Millimeter wave radar can be used as an independent system or as a part of a hybrid remote sensing implementation, which involves optical (visible or infra-red) sensors. Such a scheme can also be installed in vehicles (including autonomous ones) to distinguish between targets and adjust the system’s response to the perceived target. In addition, this technique can be used for security purposes and allow the identification of hostile activity in a limited distance, during bad weather conditions (heavy fog or haze), when sensors operating in optical wavelengths are useless.

The presented scheme can be further developed to be integrated with different sensors to assemble a compact, low-cost system. Using a quadrature detection scheme will improve performance and enables identification of movement direction.

## Figures and Tables

**Table 1:** The results of the pendulum length estimation obtained from the physical pendulum experiment.

Pendulum Measured Length *L* (m)	Initial Angle $\psi_{\max}$ (Degrees)	Maximum Velocity $v_{\max}$ (m/s)	Temporal Period *T* (s)	Calculated Pendulum Length *L* (m)	Length Error ΔL (%)	Length Uncertainty δL (m)
2.94	10	0.965	3.031	2.67	9	0.28
2.94	20	1.899	3.031	2.63	11	0.15
2.04	10	0.903	2.439	20.1	1.5	0.23
2.04	20	1.776	2.439	1.98	3.2	0.11
1.53	10	0.748	2.128	1.45	5.1	0.17
1.53	20	1.433	2.128	1.39	9.1	0.09

**Table 2:** Results of adult and child height estimation obtained from the human motion experiment.

		Human Height *H*-Measured (m)	Initial Angle $\psi_{\max}$ (Degrees)	Maximum Velocity $v_{\max}$ (m/s)	Temporal Period *T* (s)	Human Height *H*-Calculated (m)	Height Error ΔH (%)	Height Uncertainty δH (m)
Adult	Leg	1.72	30	2.5	1.266	1.81	5.5	0.16
	Hand	1.72	30	1.8	1.266	1.57	8.5	0.12
Child	Leg	1.25	30	2.7	0.833	1.29	3.2	0.04
	Hand	1.25	30	2.1	0.833	1.21	3.3	0.03
